# Analysis of the Influence of Farmer Behavior on Heavy Metal Pollution in Farmland Soil: A Case Study of Shouyang County, Shanxi Province

**DOI:** 10.3390/toxics13121040

**Published:** 2025-11-30

**Authors:** Jin-Xian Han, Yu-Jiao Liang, Feng-Mei Ban

**Affiliations:** 1 Department of Resources and Environment, Shanxi University of Finance and Economics, Taiyuan 030006, China; hanjinxian2009@163.com (J.-X.H.); liangyujiao1005@163.com (Y.-J.L.); 2 Department of Resources and Environment, Hubei University, Wuhan 430062, China

**Keywords:** farmer behavior, heavy metal content, farmland soil, geographical detector, pollution assessment

## Abstract

Building upon a theoretical framework, this study utilized 126 field survey questionnaires from farmers in Shouyang County, Shanxi Province, China, coupled with corresponding farmland soil heavy metal monitoring data, to investigate the extent of heavy metal pollution and its mechanistic relationship with farmers’ behavior. The single-factor pollution index (*P_i_*), Nemerow composite pollution index (*P_N_*), and geographical detector were employed to assess pollution levels and elucidate the underlying mechanisms linking farmer practices to soil heavy metal accumulation. Analysis revealed that the mean concentrations of Cu, Ni, Cr, Pb, Cd, and Zn (25.54, 31.47, 98.50, 16.63, 0.16 and 76.92 mg/kg, respectively) in the farmland soil exceeded the background values for soil elements in Shanxi Province, whereas As (1.92 mg/kg) levels were lower. Assessment using *P_i_* indicated that Cr, Pb, Cd, Ni, Cu, and Zn (1.78, 1.13, 1.55, 1.05, 1.07 and 1.21, respectively) were predominantly in a state of mild pollution. Similarly, the *P_N_* (1.50) suggested an overall mild level of composite heavy metal pollution in the soil. Geographical detector(Geo-Detector) analysis demonstrated that the explanatory power (q-value) of interactions among factors-including agricultural film and fertilizer application intensity, farmland fragmentation degree, per capita annual household income, farmland area, and years engaged in farming-on soil heavy metal accumulation was significantly enhanced compared to that of individual behavioral factors. While individual farmers’ behaviors are associated with heavy metal accumulation, the interaction effects among multiple behaviors constitute the dominant factor influencing localized accumulation in farmland soil. Consequently, local authorities should enhance farmers’ requisite knowledge, skills, and practices for mitigating soil heavy metal accumulation through strategies such as promoting large-scale farming, implementing agricultural input reduction initiatives, and intensifying technical and environmental protection training. The Geo-Detector exhibits significant advantages in identifying nonlinear influencing factors and analyzing factor interactions, yielding more comprehensive insights compared to conventional linear models.

## 1. Introduction

Soil is a vital resource that underpins human survival. In recent years, escalating soil pollution, particularly contamination by heavy metals (HMs), has become a critical environmental concern [1,2]. In recent years, the problem of soil pollution has been increasingly intensifying, among which HM pollution is particularly prominent; the global situation of soil HM pollution is even more severe—there are currently over 10 million contaminated sites, covering an area of 200,000 km^2^, and more than 50% of these contaminated sites are caused by HM pollution. The issue of HM pollution in farmland in China is also highly prominent: according to the first survey report on soil pollution in China, the exceedance rates of Cd, Hg, As, Cu, Pb, Cr, Zn, and Ni8 heavy metal pollution points in farmland soil were 7.0%, 1.6%, 2.7%, 2.1%, 1.5%, 1.1%, 0.9%, and 4.8%, respectively [3]. HM pollution in soil not only impairs the core functions of soil and inhibits crop growth, but also poses a serious threat to human health through the food chain [4,5]. Therefore, the remediation of HM pollution in agricultural soil in China has become an important task that urgently needs to be advanced.

The HM content in agricultural soil is affected not only by inherent natural factors such as parent soil materials, but also by external anthropogenic factors. Among these, human activities—particularly the agricultural production and management behaviors of farmers—have become the primary and direct inducement of agricultural soil HM pollution [3,6,7]. It is thus of great significance to clarify the influence mechanism of farmers’ behaviors on agricultural soil HM pollution: this is not only the key to fulfilling the requirement of “strengthening the source tracing and remediation of agricultural soil HM pollution” specified in core policies such as the 2025 Central No.1 Document, but can also provide support for the government to formulate scientific green development policies, safeguard human health, and advance the all-round revitalization of rural areas.

Existing studies have confirmed that farmers’ behaviors exert a significant impact on the HM content in agricultural soil. Specifically, irrigation methods [8,9], land use patterns [10,11,12], the adoption of agricultural technologies and household income levels [13,14], as well as field management measures and crop selection [15], have all been proven to be important factors contributing to soil HM accumulation. In research related to pollution remediation, scholars have also found that reducing soil HM content effectively can be achieved by changing farmers’ behaviors or implementing specific management measures, including adjusting planting structures [16], adopting formulated fertilization [17], conducting deep tillage, adopting phytoremediation [18], improving farmers’ educational levels [19], and increasing household income [20,21]. Although these studies provide valuable insights from diverse perspectives, many remain descriptive or qualitative. Quantitative analyses specifically focusing on farmers’ behaviors as the primary lens are scarce, with the notable exception of Ren et al. [3]. This study adopted the stepwise regression method to analyze the impact of individual farmers’ behaviors on soil HM content.

However, this method has inherent limitations: it ignores the potential combined effects of multiple farmers’ behaviors and fails to capture the nonlinear relationships and interaction effects among various farmers’ behaviors. It is precisely these relationships and effects that jointly determine the process of soil HM accumulation. In contrast, the Geo-Detector is not only proficient in analyzing linear and nonlinear correlations between variables, but also capable of quantitatively measuring the degree of interactive influence among multiple variables [7,10]. This advantage makes it a powerful tool for exploring the mechanism underlying the impact of the interactive effects of multiple farmers’ behaviors on soil heavy metal content, facilitating the acquisition of more comprehensive and systematic research conclusions.

Therefore, this study, grounded in a constructed theoretical framework and employing field sampling, household surveys, and laboratory analysis in Pingtou Town, Shouyang County, Shanxi Province, aims to: (1) evaluate the pollution status of heavy metals (Cr, As, Pb, Cd, Ni, Cu, Zn) in cultivated soil using pollution index methods; and (2) utilize the Geo-Detector to quantitatively address the core scientific question: “Which specific farmers’ behaviors and their interactions influence soil HM content, and to what extent, within an area of relatively consistent natural geographical conditions?”. The findings seek to clarify the relationship between farmers’ behaviors and HM accumulation in farmland soil, identify quantitatively the single and interactive factors that influence the accumulation of heavy metals in local soil, and provide a decision-making reference for formulating precise regional pollution mitigation strategies. Meanwhile, the findings also seek to provide a new perspective for subsequent quantitative analysis of heavy metal accumulation factors.

## 2. Materials and Methods

### 2.1. Theoretical Framework

**Figure 1:**
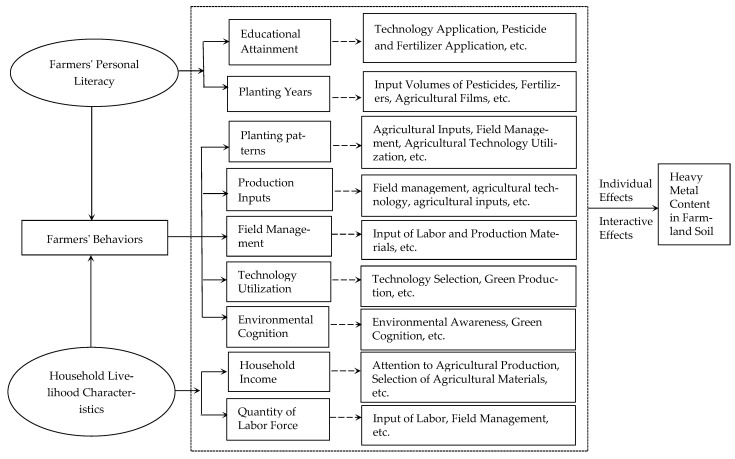
Theoretical analysis framework of the impact of farmers’ behaviors on heavy metal content in farmland soil.

Farmers, as the primary agents of China’s rural economy, function as the direct managers and users of agricultural land. Amidst growing concerns over agricultural pollution, the influence of farmers’ behaviors on environmental issues has garnered significant academic attention. Heavy metal (HM) pollution in farmland soil stands out as one of the most pressing environmental challenges within Chinese agriculture [3]. Shaped by their individual literacy and household characteristics within specific socio-economic contexts, farmers may overlook the environmental ramifications of their production practices. Substantial evidence identifies farmers’ behaviors as a primary driver of HM pollution in cultivated soil. These behaviors encompass a spectrum of activities: planting decisions (patterns and crop varieties), production input selection, technology adoption, field management, and resource utilization (Figure 1). Specifically, choices regarding planting patterns and crop varieties can modify agricultural landscapes, input regimes, and management practices, subsequently influencing soil HM content [15,16]. Input behaviors (e.g., fertilizer, pesticide, film application) and field management practices directly affect HM levels in soil [9,11,17]. Furthermore, factors like environmental awareness, conservation attitudes, technology adoption, farmland fragmentation, and household income directly or indirectly modulate agricultural input intensity, potentially triggering or exacerbating HM pollution [3,19].

Building upon this theoretical foundation, we constructed an analytical framework delineating the impact pathways of farmers’ behaviors on farmland soil HM content (Figure 1). The core proposition is that within regions characterized by consistent natural geographical conditions, farmers’ behaviors—including agricultural input use, technology adoption, cropping patterns, and environmental cognition—shaped by personal literacy and household characteristics exert both individual and interactive effects on soil HM content, leading to accumulation. To operationalize this framework, the aforementioned behaviors were refined into specific measurable factors. Corresponding evaluation indicators were selected (Table 1), a structured farmer questionnaire was designed, and concurrent field surveys and soil samples collection were implemented.

### 2.2. Data Sources

#### 2.2.1. Selection of Study Area and Samples

The study area, Pingtou Town, Shouyang County, Shanxi Province (112°49′–112°56′ E, 37°55′–37°58′ N), has a flat terrain and belongs to the Quaternary loess sedimentary zone and the warm temperate semi-arid continental monsoon climate zone. The annual average precipitation is 570 mm; the average annual temperature is 7.4 °C, and the sunshine hours are 2725 h. The spring is dry and windy, the summer is hot and rainy, the autumn is clear and pleasant, and the winter is cold with little snow. The spring and autumn seasons are short, while the winter and summer seasons are relatively long. The soil type is light brown soil, with an average pH value of 8.0. The study area possesses a long history of cultivation, with primary food crops including corn and millet, and is a typical dryland conventional agricultural area. Conventional agriculture is a stage in the process of transforming traditional agriculture into modern agriculture. The extensive use of various resources, including pesticides, fertilizers, agricultural films, expanders, herbicides, etc., is an important characteristic of conventional agriculture, and the input of these resources is often closely related to farmers. Since the 1990s, vegetable cultivation (e.g., cabbage, zucchini, and radish) has been introduced, establishing a dominant cropping structure of corn and vegetables, featuring patterns such as continuous corn monoculture and vegetable–corn rotation. This diversity provides comparable samples for analyzing HM accumulation in farmland soil under different management regimes.

To specifically examine the impact of farmers’ behaviors on farmland soil HM pollution, semi-structured questionnaires were administered to farmers owning the sampled plots concurrently with soil sampling, supplemented by in-depth face-to-face interviews. A total of 130 questionnaires were distributed and 130 paired soil samples were collected. Ultimately, 126 valid questionnaires and corresponding soil samples were obtained, yielding an effective response rate of 96.92%. The questionnaire encompassed: basic characteristics of household heads (X_1_–X_4_), household livelihood characteristics (X_5_–X_8_), and cultivated land resource characteristics (X_9_–X_10_), agricultural input behaviors (X_11_–X_14_), environmental cognition levels (X_15_–X_16_), farmers’ technical proficiency (X_17_), and cropping patterns (X_18_). Table 1 details the selection, assignment, and descriptive statistics of these farmers’ behavioral variables, reflecting the actual conditions of the study area.

#### 2.2.2. Soil Sample Collection and Analysis

**Figure 2:**
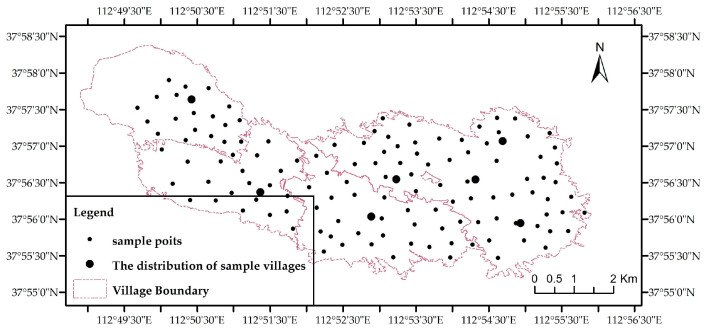
Schematic diagram of the study area and surveyed villages.

Each sampling site encompassed approximately 0.067 hm^2^ (1 mu) and was situated away from roads and residential areas, with no factories and mining enterprises in the surrounding area. Furthermore, natural factors (climate, topography—relatively flat, parent material) within the study area were relatively homogeneous, minimizing the confounding effects of natural variation and surrounding environments on farmland soil HM content. Within each plot, five sub-samples were collected from the topsoil layer (0–20 cm depth) along an S-shaped path (serpentine sampling). These sub-samples were homogenized to form a composite sample, sealed in polyvinyl chloride (PVC) bags, labeled, and transported to the laboratory for analysis. Soil samples (n = 126) were collected across 7 villages. The geographical distribution of the study area and surveyed villages is depicted in Figure 2.

All samples were naturally air-dried in the laboratory, with impurities removed. 50 g of the sample was crushed and passed through a 2 mm nylon sieve. The mixture was then thoroughly mixed and further ground using an agate mortar to pass through a 0.149 mm nylon sieve. The sample was then stored for testing.

Approximately 0.1000 g of the ground soil sample was weighed using an electronic balance. The sample was digested via the “HNO_3_-HF-HClO_4_” method, and the contents of Cr, Pb, Cu, and Ni in the soil sample were determined using an inductively coupled plasma mass spectrometer (X-Series ICP-MS, Thermo Fisher Scientific, Waltham, MA, USA), in accordance with GB/T 17137-1997 [22], GB/T 17141-1997 [23], GB/T 17138-1997 [24], and GB/T 17139-1997 [25]. The contents of Cd and Zn in the soil sample were determined using an atomic absorption spectrophotometer (Agilent 240FS-AA, Agilent Technologies, Santa Clara, CA, USA), following GB/T 17141-1997 [23] and GB/T 17138-1997 [24].

Approximately 0.2000 g of the ground soil sample was weighed, and the sample was digested with aqua regia in a water bath. The contents of As in the soil sample was determined by atomic fluorescence spectrometry (GB/T 22105.2-2008) [26] using a Beijing Haiguang KYS02 atomic fluorescence photometer (Beijing Haiguang Instrument Co., Ltd., Beijing, China) [27].

During the determination process, all reagents used were of guaranteed reagent grade. All samples were analyzed in 2 parallel replicates, and recovery tests were conducted using national standard soil samples GSS-8. The relative standard error of the test results of national standard soil samples is less than 10%, confirming the accuracy and reliability of all soil sample test results.

### 2.3. Methods

#### 2.3.1. Soil Heavy Metal Pollution Index 

Pollution index methods are widely employed for assessing soil HM pollution. The single-factor pollution index (*P_i_*) evaluates the contamination level of individual HMs. Conversely, the Nemerow composite pollution index (*P_N_*) provides a holistic assessment of overall regional soil HM pollution, emphasizing the contribution of the most severely contaminated metals [3,28].

Single-factor pollution index:

(1) $$P_{i}\;={\;C}_{i}/C_{n}$$

In the formula: $P_{i}$ is the pollution index of heavy metal *i* in the soil, $C_{i}$ is the measured concentration of heavy metal *i* (mg/kg), and $C_{n}$ is the standard limit value of heavy metal *i* (mg/kg). In this study, the background values of soil elements in Shanxi Province were adopted (Table 2). The classification standards are as follows: When $P_{i}$ ≤ 0.7, the soil environmental quality is classified as clean and unpolluted; When 0.7 < $P_{i}$ ≤ 1.0, it is alert and still clean; When 1.0 < $P_{i}$ ≤ 2.0, it is mildly polluted; When 2.0 < $P_{i}$ ≤ 3.0, it is moderately polluted; When $P_{i}$ > 3.0, it is heavily polluted [27].

Nemerow composite pollution index:

(2) $$P_{N}=\sqrt{\left({P_{\mathrm{iave}}}^{2}+{P_{\mathrm{imax}}}^{2}\right)/2}$$

In the formula: $P_{N}$ is the composite pollution index of multiple heavy metals in the soil; $P_{\mathrm{iave}}$ is the average value of the single heavy metal pollution indices in the soil; $P_{\mathrm{imax}}$ is the maximum value of the single heavy metal pollution indices in the soil. The classification standards for $P_{N}$ are as follows: $P_{N}$ ≤ 0.7 indicates safe and clean; 0.7 < $P_{N}$ ≤ 1.0 indicates alert and still clean; 1.0 < $P_{N}$ ≤ 2.0 indicates mild pollution; 2.0 < $P_{N}$ ≤ 3.0 indicates moderate pollution; and $P_{N}$ > 3.0 indicates heavy pollution [28].

#### 2.3.2. Geo-Detector for the Impact of Farmers’ Behaviors on Heavy Metal Pollution in Farmland Soil

The Geo-Detector model is a statistical toolkit designed to detect spatial heterogeneity and identify its underlying drivers [29]. It accommodates both numerical and categorical data without assuming linear relationships between variables. Notably, it exhibits superior statistical accuracy compared to other models when sample sizes are small (n < 30) [29]. Consequently, it has been successfully applied to analyze geographical environmental factors, including the detection of drivers of soil HM pollution [7,10,30,31]. However, its application specifically to farmer behavior influencing soil HM pollution remains limited. The Geo-Detector comprises four modules: factor detector, risk detector, ecological detector, and interaction detector. This study primarily employed the factor detector and interaction detector to investigate the influence of farmers’ behaviors (*X*_1_–*X*_18_, Table 1) on the spatial differentiation of soil HM pollution within the study area and to identify the primary drivers.

(1) Factor detector: The factor detector quantifies the influence of each independent variable on the spatial heterogeneity of the dependent variable (here, soil HM pollution) using the q-statistic. The q-value ranges from 0 to 1, where a higher value indicates greater explanatory power of the factor over the spatial pattern of HM pollution, signifying a stronger influence. Primary influencing factors were identified based on higher q-values. The q-statistic is calculated as [29]:
  (3) $$q=1-\frac{\sum_{h=1}^{L}N_{h}\sigma_{h}^{2}}{N\sigma^{2}}=1-\frac{\mathrm{SSW}}{\mathrm{SST}}\;\;\mathrm{SSW}=\sum_{h=1}^{L}N_{h}\sigma_{h}^{2}\mathrm{SST}=N\sigma^{2}$$
  In the formula: q represents the explanatory power of the factor; h = 1, …, L denotes the stratification or classification of variable Y or factor X; N_h_ and N are the number of units in the stratum and the entire region, respectively; σ_h_^2^ and σ^2^ are the variances of Y-values in the stratum and the entire region, respectively; and SSW are the total within-stratum variance and total regional variance, respectively.
(2) Interaction detector: The interaction detector identifies the type and magnitude of influence arising from interactions between two independent variables by comparing their individual q-values with the q-value of their interaction (q (X_1_ ∩ X_2_)). A higher q (X_1_ ∩ X_2_) indicates stronger combined explanatory power, aiding in identifying synergistic factors driving soil HM pollution. The specific interaction types and their determination criteria are detailed in Table 3.
(3) Data type conversion: A critical prerequisite for geographic detector analysis is that all independent variables must be categorical in nature. When continuous variables are encountered in the dataset, the software will systematically generate an error during execution. To address this issue, several established data discretization methods are commonly employed: natural breaks classification, equal interval classification, quantile classification, and manual classification. Each methodology exhibits distinct optimal applications based on data characteristics.

**Table 3:** Types and criteria of interaction between factors.

Types of Interaction	Criteria
Bivariate enhancement	q(X_1_ ∩ X_2_) > Max(q(X_1_), q(X_2_))
Nonlinear enhancement	q(X_1_ ∩ X _2_) > (q(X_1_) + q(X_2_)
Independence	q(X_1_ ∩ X_2_) = q(X_1_) + q(X_2_)
Nonlinear weakening	q(X_1_ ∩ X_2_) < Min(q(X_1_), q(X_2_))
Single-factor nonlinear weakening	Min(q(X_1_), q(X_2_)) < q(X_1_ ∩ X_2_) < Max(q(X_1_), q(X_2_))

The natural breaks classification method is particularly advantageous as a preliminary approach when the underlying data distribution patterns remain unclear. For datasets characterized by a relatively constrained range and minimal extreme values, the equal interval classification method proves most appropriate. In contrast, quantile classification is specifically designed for data demonstrating linear distribution patterns. Manual classification should be prioritized when industry standards dictate fixed segmentation intervals, as exemplified in population density distribution grading systems.

In the present study, considering the distribution characteristics of survey indicators, including X_1_, X_5_, X_7_, X_8_, X_9_, X_10_, X_11_, X_12_, and X_13_, the equal interval classification method was systematically implemented to convert these continuous variables into categorical quantities suitable for geographic detector analysis.

## 3. Results and Analysis

### 3.1. Statistical Results of the Questionnaire Survey

**Figure 3:**
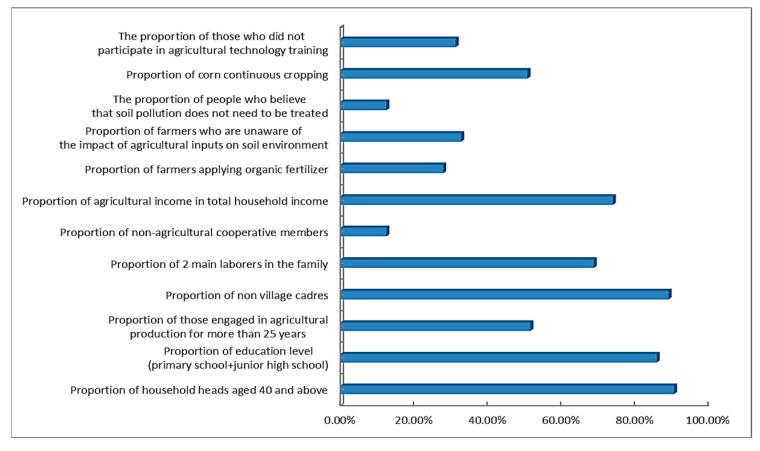
Statistical Results of the Questionnaire Survey in the study area.

Questionnaire surveys (Figure 3) indicate that the average age of household heads engaged in agricultural production in the study area is 52 years, with 90.6% of them being over 40 years old. Furthermore, the educational level of these household heads is mainly primary and junior high school, which aligns with the common characteristic of rural China, where agricultural practitioners are predominantly middle-aged and elderly with relatively low educational attainment.

The questionnaire surveys (Table 1) also reveal that the annual household income of farmers is relatively low, and agricultural income remains the primary source of income for local farmers. The input of agricultural resources such as fertilizers, pesticides, and agricultural films is still the main means for local farmers to increase crop yields. Moreover, constrained by traditional farming concepts and educational levels, a large proportion of farmers in the study area have not participated in agricultural technology training. A relatively high percentage of farmers believe that the input of agricultural resources has no impact on soil pollution, and some further hold the view that soil pollution does not require treatment (Figure 3). At the same time, the large-scale cultivation is still in its initial stage in the study area, which, to a certain extent, results in a high degree of cultivated land fragmentation (mean value is 2.4 mu/plot) and increased labor and production costs. For instance, 5 mu of land owned by some farmers is divided into 7 small plots.

These characteristics and behaviors of farmers could be potential sources of risk for soil environmental pollution.

### 3.2. Statistical Characteristics of Soil Heavy Metal Contents

As presented in Table 2, the mean concentrations of HMs in the study area’s farmland soil followed the sequence: Cr > Zn > Ni > Cu > Pb > As > Cd. The mean concentrations of Cu, Ni, Cr, Pb, Cd, and Zn exceeded their respective background values for Shanxi Province, whereas As was lower. The magnitude of exceedance relative to background values ranked as: Cr (0.78) > Cd (0.55) > Zn (0.21) > Pb (0.13) > Cu (0.07) > Ni (0.05). This confirms the occurrence of HM accumulation in the study area’s farmland soil. Coefficients of variation (CV) indicated moderate to high variability in soil HM contents, with no low variability (CV< 16%). Specifically, Ni, Cr, Pb, and Cd exhibited moderate variability (16% ≤ CV < 36%), while As and Zn displayed high variability (CV ≥ 36%). Higher CV values suggest greater spatial heterogeneity in soil HM distribution, implying a stronger influence from localized exogenous inputs, such as anthropogenic activities [28], which warrants attention.

### 3.3. Assessment of Soil Heavy Metal Pollution

**Figure 4:**
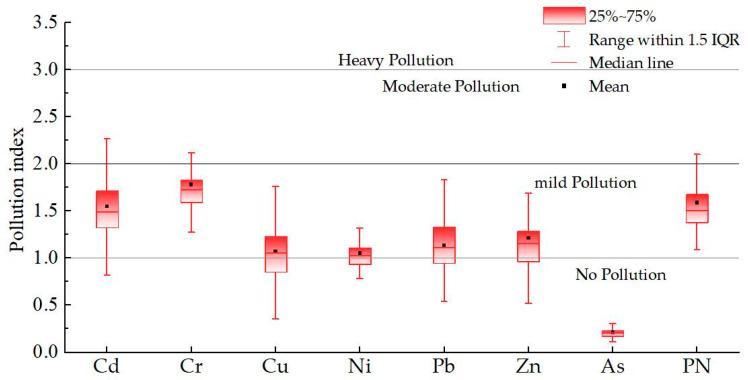
Pollution assessment results of soil heavy metal pollution in the study area.

**Figure 5:**
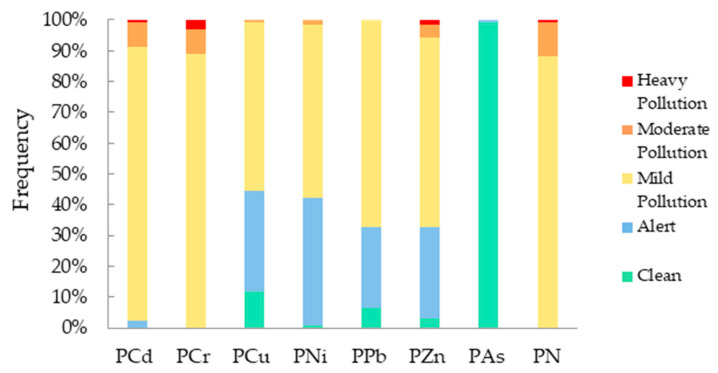
Frequency distribution of soil heavy metal pollution index in different pollution levels in the study area.

Results of the single-factor pollution index (*P_i_*) assessment are summarized in Figure 4. The mean *P_i_* values for the seven HMs were: As (0.21), Ni (1.05), Cu (1.07), Pb (1.13), Zn (1.21), Cd (1.55), and Cr (1.78). This indicates that, with the exception of As, all other HMs exhibited varying degrees of exceedance above background values within the study area’s farmland soil. Based on the *P_i_* classification criteria, As was classified as clean/unpolluted, while Ni, Cu, Cr, Pb, Cd, and Zn were categorized as mildly polluted. The frequency distribution of *P_i_* values across pollution levels provides further detail on the accumulation status of each HM (Figure 5).

The Nemerow composite pollution index (*P_N_*) evaluation results are also shown in Figure 4. The mean *P_N_* for the study area was 1.50, indicating an overall mild pollution level. Nevertheless, one sample was classified as severely polluted and 14 as moderately polluted. The spatial distribution of composite HM pollution across different severity grades is detailed in Figure 5.

### 3.4. Spatial Distribution of Soil Heavy Metal Pollution

**Figure 6:**
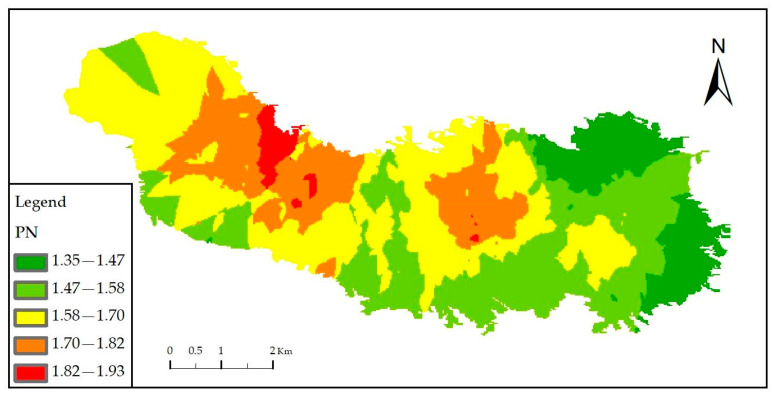
Spatial distribution of the Nemerow composite pollution index (*P_N_*) in the study area.

Spatial interpolation of the composite heavy metal pollution index of soil at sampling points can intuitively reflect the spatial heterogeneity of soil heavy metal pollution. Among the geostatistical techniques available, Kriging interpolation and Inverse Distance Weighting (IDW) interpolation are the most widely employed methods for spatial prediction. In this study, we adopted Ordinary Kriging (OK) as the primary interpolation method, supported by rigorous statistical validation. The Kolmogorov–Smirnov (K-S) test conducted in SPSS (version 26.0) confirmed that the *P_N_* values for the seven heavy metals exhibited a normal distribution (*p* < 0.01, Sig. = 0.000). This statistical finding justified the application of Ordinary Kriging, which assumes normally distributed data, for generating accurate spatial distribution maps. Therefore, with the support of ArcMap 10.0, ordinary Kriging interpolation was employed in this study to generate the spatial distribution map of *P_N_* in the study area (Figure 6).

It can be observed from the map (Figure 6) that there is distinct spatial heterogeneity in the comprehensive pollution of the 7 heavy metals in the farmland soil of the study area, with the pollution level decreasing from the peak areas to the surrounding regions. The soil heavy metal pollution in the peak areas may pose risks to local agricultural products and residents’ health, which should be paid close attention to.

### 3.5. Geo-Detector Analysis of the Impact of Farmers’ Behaviors on Heavy Metal Pollution in Farmland Soil

**Figure 7:**
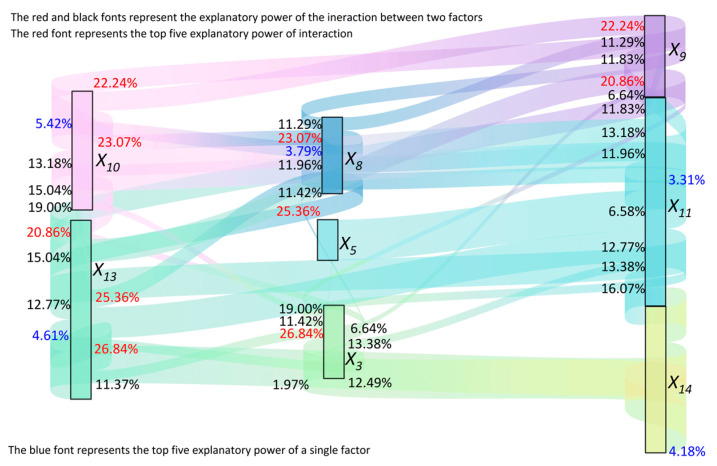
Sankey diagram of the explanatory power of farmers’ behavior on heavy metal pollution in farmland soil.

Spatial heterogeneity in HM accumulation was observed (as indicated by Figure 6) across the above research in the study area. The Geo-Detector operates on the principle that if an independent variable significantly influences a dependent variable, their spatial distributions should exhibit similarity [29]. This principle allows the Geo-Detector to identify factors driving the spatial differentiation of the dependent variable and reveal which independent variables exert significant influences. Therefore, the HM composite pollution index (*P_N_*) served as the dependent variable, and two sub-models (the single-factor detector and interaction detector) were applied to quantitatively assess the explanatory power (q-value) of farmers’ behaviors (Table 1) on soil HM accumulation, thereby testing the theoretical framework. The top five influencing factors based on individual q-values, along with the top five two-factor interactions based on interaction q-values, were visualized using a Sankey diagram (generated with Origin 2024), presented in Figure 7.

The Sankey diagram depicting interaction effects (Figure 7) reveals that the q-values for interactions between any two farmer behavior factors exceeded those of the individual factors alone. Based on the criteria in Table 2, all observed interaction types were classified as “nonlinear enhancement,” signifying that the q-value for the interaction (q (*X*_1_∩*X*_2_)) was greater than the sum of the q-values of the individual factors (q(*X*_1_) + q(*X*_2_)). This demonstrates that the combined effect of interacting behavioral factors on local soil HM accumulation surpasses the impact of individual factors. Specifically, the top five two-factor interactions exhibiting the strongest explanatory power for soil composite HM pollution (*P_N_*) were: Agricultural film application intensity (*X*_13_) ∩ Years of agricultural production (*X*_3_), Per capita annual household income (*X*_8_) ∩ Agricultural film application intensity (*X*_13_), Per capita annual household income (*X*_8_) ∩ Farmland fragmentation degree (*X*_10_), Farmland area (*X*_9_) ∩ Farmland fragmentation degree (*X*_10_), and Farmland area (*X*_9_) ∩ Agricultural film application intensity (*X*_13_).

Concurrently, the top five individual farmers’ behaviors ranked by their q-value (explanatory power) for local soil HM accumulation were (Figure 7): Farmland fragmentation degree (*X*_10_) > Agricultural film application intensity (*X*_13_) > Organic fertilizer application (*X*_14_) > Per capita annual household income (X_8_) > Chemical fertilizer application intensity (*X*_11_). This indicates that farmland fragmentation, agricultural film application intensity, per capita annual household income, organic fertilizer use, and chemical fertilizer application intensity significantly influence HM accumulation in the study area. Conversely, other factors, including crop planting pattern selection (*X*_18_), exhibited negligible impact, potentially attributable to limited variation in farmer responses regarding these specific factors within the sampled population.

## 4. Discussion

### 4.1. Characteristics and Causes of Heavy Metal Accumulation in Farmland Soil of the Study Area

Soil serves as the fundamental resource for agricultural production, and its quality is intrinsically linked to the safety and quality of agricultural products, furthermore human health. In China, farmers function as the primary operators and stewards of rural land. Given the escalating prevalence of agricultural pollution, the influence of farmers’ behaviors on environmental degradation has emerged as a critical area of scholarly and policy concern. Among the various environmental challenges, heavy metal contamination in arable soils represents one of the most intractable issues confronting China’s agricultural ecosystem. Numerous studies acknowledge farmer behavior as a major contributor to farmland soil HM pollution. However, existing research predominantly offers descriptive or qualitative discussions on this relationship. Quantitative analyses specifically focusing on farmer behavior impacts remain limited, with the notable exception of Ren et al. [3], who employed regression models to assess the influence of individual behaviors. Building upon prior research, this study, conducted in Shouyang County, Shanxi Province, collected data within an area of relatively homogeneous natural conditions. It employed the Geo-Detector to quantitatively analyze the impact of both individual farmers’ behaviors and their interactions on soil HM content, thereby enriching the relevant literature.

Our investigation confirmed that concentrations of As, Cu, Ni, Cr, Pb, Cd, and Zn in the study area did not exceed the risk control thresholds stipulated in the Soil Environmental Quality Risk Control Standard for Soil Contamination of Agricultural Land (GB 15618-2018) [32]. However, assessment using *P_i_* relative to Shanxi Province background values revealed that, except for As (clean/unpolluted), Ni, Cu, Cr, Pb, Cd, and Zn were in a state of mild pollution. This signifies varying degrees of accumulation for these six HMs, with Cr and Cd exhibiting particularly pronounced accumulation. This accumulation arises from a combination of exogenous inputs, including farmer practices, and the intrinsic biogeochemical behavior of different HMs within the soil matrix. Local agricultural practices rely heavily on inputs such as agricultural film, chemical fertilizers, pesticides, herbicides, and organic fertilizers to enhance productivity. These inputs are recognized sources of HMs like Cd, Cr, Cu, and Zn [31,32,33,34,35]. Notably, there is a local practice of applying chicken manure as organic fertilizer, and studies indicate its Cr content surpasses that of manure from livestock (e.g., cattle, sheep) or other poultry (e.g., pigs, geese) [33,34]. Collectively, these practices elevate the risk of HM accumulation. Additionally, the alkaline soil conditions (mean pH = 8.0) prevalent in the study area promote the adsorption and fixation of Cr and Cd by soil colloids, limiting their migration and leading to their retention and accumulation in the surface soil horizon [36,37].

From the spatial distribution map of the comprehensive pollution index (*P_N_*) in the study area (Figure 6), it can be seen that the soil heavy metal contents in this region exhibit certain spatial heterogeneity. However, the study area has a small scope, with uniform soil texture and limited differences in parent materials. Moreover, the impacts of human activities such as transportation and industrial activities were avoided during sampling. Therefore, farmers’ behaviors may be the main factor contributing to the spatial heterogeneity in soil heavy metal accumulation.

In addition, a study by Yan Jiao et al. on the causes of village-level soil heavy metal pollution also shows that under the condition of consistent local natural conditions, differences in farmers’ production behaviors on different plots are more likely to lead to the local accumulation of soil heavy metals [38].

### 4.2. Impact of Farmers’ Behaviors on Heavy Metal Accumulation in Farmland Soil of the Study Area

Results from the single-factor detection of the Geo-Detector identified farmland fragmentation degree (*X*_10_), agricultural film application intensity (*X*_13_), per capita annual household income (*X*_8_), organic fertilizer application (X_14_), and chemical fertilizer application intensity (*X*_11_) as exhibiting relatively high explanatory power (q-value) for composite soil HM pollution (*P_N_*). This signifies a significant influence of these individual farmers’ behaviors on HM accumulation within the study area. Field investigations confirmed farmland fragmentation as a prominent feature of local agriculture. Fragmentation elevates production costs (e.g., labor, fertilizer) [39,40,41] and potentially increases the application intensity of HM-containing inputs per unit area managed. Farmers with higher household income levels primarily rely on off-farm or migratory work, potentially leading to reduced attention to agricultural production. This may manifest in suboptimal choices regarding the quality and quantity of agricultural inputs. Agricultural film, essential for local dryland farming, often contains Cd, Pb, and Zn additives (e.g., heat stabilizers) introduced during production. Furthermore, residual film particles can act as vectors for HM enrichment in soil [42]. Both chemical fertilizers and organic fertilizers are recognized sources of HMs like Cd, Cr, Pb, Cu, Zn, and Ni [33,34,35]. Cumulatively, these factors associated with specific farmers’ behaviors contribute to the elevated risk of HM pollution.

The interaction detector results demonstrated that the impact (quantified by q-value) of interactions between any two farmer behavior factors on composite HM pollution (*P_N_*) was significantly stronger than the impact of any single factor alone. This highlights that interactions among behavioral factors substantially enhance the explanatory power for soil HM pollution, corroborating findings from studies utilizing the Geo-Detector in other environmental contexts [7,10,28,30]. Among them, the interaction impact between agricultural film application intensity (X_13_) and years of engaging in agricultural production (X_3_) is the most prominent, which can be attributed to the fact that prolonged planting duration leads to increased residual agricultural film accumulation in the soil. Additionally, the interaction impact between per capita annual household income (X_8_) and agricultural film application intensity (X_13_), as well as the degree of farmland fragmentation (X_10_), exhibited notable prominence. Empirical research has revealed that households with higher local income levels typically demonstrate a diminished proportion of agricultural income and reduced engagement in agricultural production activities [3,39]. Furthermore, due to the inherent fragmentation of farmland, agricultural producers often resort to increased agricultural film usage as a labor-saving strategy to mitigate time-intensive field management tasks, such as weeding. The interaction impact between the household farmland area (X_9_) and the farmland fragmentation degree (X_10_), as well as agricultural film application intensity (X_13_), also proved more significant. On one hand, this phenomenon may stem from the initial stage of intensive arable land utilization in the region, where larger land areas are associated with higher degrees of fragmentation. On the other hand, it could be related to the increased investment in agricultural film necessitated by larger and more dispersed farmland areas [3,41]. Combining the results of the interaction detection (Figure 7) and the classification of farmer behavior (Table 1), it is evident that interactions between agricultural input behaviors (*X*_11_–*X*_14_), farmland resource characteristics (*X_9_*–*X*_10_), household livelihood characteristics (*X*_5_–*X*_8_), and household head basic characteristics (*X*_1_–*X*_4_) significantly contribute to soil HM pollution. This underscores that the interplay among these diverse farmers’ behaviors constitutes the dominant driver of localized soil HM accumulation, thereby validating the proposed theoretical framework.

### 4.3. Comparison Between Geo-Detector and Stepwise Regression Model

Stepwise regression is another common method for analyzing influencing factors [3]. For comparative purposes, stepwise regression was performed using SPSS 19.0, with the composite HM pollution index (*P_N_*) as the dependent variable and farmer behavior variables (*X*_1_*–X*_18_) as independent variables. The results are summarized in Table 4. A comparison between the Geo-Detector results (Figure 7, individual factor q-values) and the stepwise regression model (Table 4) shows congruence in identifying *X*_10_ (farmland fragmentation degree), *X*_11_(chemical fertilizer application intensity), and *X*_14_ (organic fertilizer application) as significant factors influencing *P_N_*. However, factors identified as significant by the Geo-Detector (*X*_8_—per capita annual household income and *X*_13_—agricultural film application intensity) were not retained in the stepwise regression model. This discrepancy arises because the Geo-Detector effectively captures both linear and nonlinear relationships, whereas stepwise regression primarily identifies linear associations. Consequently, stepwise regression may fail to detect significant factors involved in nonlinear relationships. While stepwise regression corroborates some key drivers identified by the Geo-Detector, it possesses inherent limitations: it cannot reliably detect nonlinear influences and is incapable of identifying interactions between factors. In contrast, the Geo-Detector accommodates both linear and nonlinear correlations and offers distinct advantages in quantitatively analyzing factor interactions and nonlinear effects. Therefore, the Geo-Detector yields a more comprehensive analytical outcome.

## 5. Conclusions and Recommendations

Based on integrated household survey data and laboratory analyses, this study empirically investigated the impact of farmer behavior on HM pollution in farmland soil, leading to the following conclusions:

(1) Relative to Shanxi Province soil background values, Cr, Pb, Cd, Ni, Cu, and Zn (excluding As) exhibited discernible accumulation in the farmland soil of the study area, with average contents of 98.50, 16.63, 0.16, 31.47, 25.54, 76.92, and 1.92 mg/kg, respectively, warranting attention. Nevertheless, overall pollution levels were assessed as mild based on both single-factor (*P_i_*-As 0.21, Ni 1.05, Cu 1.07, Pb 1.13, Zn 1.21, Cd 1.55, Cr 1.78) and composite (*P_N_*-1.50) indices.
(2) From the perspective of the spatial distribution of the comprehensive pollution index (*P*_N_) for 7 heavy metals in the study area, there are certain high-value zones of comprehensive soil heavy metal pollution. These localized hotspots suggest that, despite relatively uniform natural conditions across the region, anthropogenic factors—particularly variations in agricultural practices—play a significant role in the differential accumulation of heavy metals at the plot level.
(3) The Geo-Detector analysis revealed that the top five individual farmers’ behaviors ranked by their q-value were: Farmland fragmentation degree (5.4%), Agricultural film application intensity (4.6%), Organic fertilizer application (4.2%), Per capita annual household income (3.8%), Chemical fertilizer application intensity (3.3%) and the top five explanatory power of farmers’ behaviors interaction ranked by their q-value were: Agricultural film application intensity ∩ Years of agricultural production(26.8%), Per capita annual household income ∩ Agricultural film application intensity(25.4%), Per capita annual household income ∩ Farmland fragmentation degree(23.1%), Farmland area ∩ Farmland fragmentation degree (22.2%), and Farmland area ∩ Agricultural film application intensity (20.9%). These revealed that for Shouyang County, Shanxi Province while individual farmers’ behaviors—specifically farmland fragmentation degree (*X*_10_), agricultural film application intensity (*X*_13_), organic fertilizer application (*X*_14_), per capita annual household income (*X*_8_), and chemical fertilizer application intensity (*X*_11_)—were significantly associated with HM accumulation, the explanatory power of interactions between any two behavioral factors significantly surpassed that of individual factors. Consequently, interactions among diverse farmers’ behaviors constitute the dominant drivers of HM accumulation in local farmland soil. Key interaction pairs involved agricultural film application intensity (*X*_13_) intersecting with years of agricultural production (*X*_3_), per capita income (*X*_8_), farmland area (*X*_9_) and fragmentation degree (*X*_10_). Therefore, formulating effective environmental policies requires a multi-faceted and coordinated strategy. Recommendations for Shouyang County, Shanxi Province include: (i) County and township agricultural departments guiding farmers towards moderate large-scale farming to reduce fragmentation; (ii) Implementing targeted campaigns to reduce excessive agricultural input usage; and (iii) Strengthening technical training and environmental protection education for farmers. These integrated measures aim to enhance farmers’ knowledge, skills, and practices essential for reducing soil HM pollution, fostering a transition towards green and sustainable modern agriculture that harmonizes agricultural product safety with rural environmental protection.
(4) The Geo-Detector proves highly suitable for analyzing correlations involving both linear and nonlinear relationships. It exhibits significant advantages over traditional linear models (like stepwise regression) in identifying nonlinear influencing factors and quantifying interactions between factors, thereby yielding more comprehensive analytical results.
(5) This study employed Geo-Detector to analyze the relationship between heavy metal accumulation in farmland soil and farmer behavior, offering a new perspective for future quantitative research on the impact of farmer behavior on soil environmental pollution. However, due to the complexity of heavy metal accumulation in soil, future similar studies should take into account the impacts of factors such as regional variations, the selection of farmer behavior indicators, types of heavy metals, and crop species. Additionally, due to the absence of historical data on local soil heavy metal elements and farmer behavior, this study lacks an investigation into the temporal changes in the influence of farmer behavior on soil heavy metal content, but it can provide a foundation for subsequent research.

## Figures and Tables

**Table 1:** Selection, assignment and descriptive statistics of variables of farmers’ behaviors.

Variables	Name	Assignment	Average Value	Standard Deviation
Basic characteristics of household heads	Age (*X*_1_)	1 = Under 32 years; 2 = 33–42 years; 3 = 43–52 years; 4 = 53–62 years; 5 = Over 62 years	3.422	0.932
	Education level (*X*_2_)	1 = Illiterate or semi-literate; 2 = Primary school; 3 = Junior high school; 4 = Senior high school and above	2.688	0.704
	Years of engaging in agricultural production (*X*_3_)	1 = 7 years; 2 = 8–17 years; 3 = 18–27 years; 4 = Over 28 years	3.406	0.931
	Whether being a village cadre (*X*_4_)	1 = Yes; 2 = No	1.894	0.124
Household livelihood characteristics	Number of main laborers in the household (*X*_5_)	Actual number of laborers (person)	1.953	0.598
	Whether being a member of agricultural cooperative (X_6_)	1 = Yes; 2 = No	1.875	0.331
	Proportion of agricultural income in total household income (*X*_7_)	Actual proportion (%)	74%	0.320
	Per capita annual household income (*X*_8_)	Per capita annual income (10,000 yuan)	1.791	0.865
Characteristics of farmland resources	Household farmland area (*X*_9_)	Actual cultivated land area (mu)	17.232	8.505
	Degree of cultivated land fragmentation (*X*_10_)	Degree of cultivated land fragmentation (plots/mu)	0.465	0.172
Agricultural input behaviors	Chemical fertilizer application intensity (*X*_11_)	Chemical fertilizer application amount per unit area (kg/mu)	97.190	15.030
	Pesticide application intensity (*X*_12_)	Pesticide application amount per unit area (L/mu)	0.344	0.197
	Plastic film application intensity (*X*_13_)	Plastic film application amount per unit area (kg/mu)	3.319	1.396
	Whether using organic fertilizer (*X*_14_)	1 = Yes; 2 = No	1.719	0.450
Environmental cognition level	Awareness of agricultural inputs’ impact on soil (*X*_15_)	1 = Aware; 2 = Unaware	1.328	0.470
	Whether soil pollution requires remediation (*X*_16_)	1 = Very necessary; 2 = Necessary; 3 = Unnecessary	1.953	0.543
Farmers’ technical proficiency	Participation in agricultural technical training (*X*_17_)	1 = No; 2 = Yes	1.641	0.480
Cropping patterns	Selection of cropping patterns (*X*_18_)	Continuous corn cropping = 1; Vegetable–corn rotation = 2	1.492	0.500

Note: Variables *X*_5_, *X*_7_, *X*_8_, *X*_9_, *X*_10_, *X*_11_, *X*_12_, and *X*_13_ were categorized and assigned values based on actual measurements.

**Table 2:** Descriptive statistics of heavy metal content in farmland soil of the study area.

Characteristic Parameter	Heavy Metal Elements						
	Cd	Cr	Cu	Ni	Pb	Zn	As
Minimum value	0.083	70.40	8.05	18.21	7.90	32.70	0.38
Maximum value	0.309	239.23	56.75	86.27	26.92	210.12	8.23
Arithmetic mean	0.158	98.50	24.54	31.47	16.63	76.92	1.92
Standard deviation	0.038	22.99	7.56	7.43	3.97	27.48	0.72
CV	24	23	31	24	24	36	38
Background values of soil elements in Shanxi Province	0.102	55.30	22.90	29.90	14.70	63.50	9.10

Note: The unit of CV is %, and the unit of heavy metal content and background value is mg/kg.

**Table 4:** Regression analysis of the influence of farmer behavior on heavy metal pollution in farmland soil.

Model	Explanatory Variable	Standardized Coefficient	Standard Error	t-Statistic	Sig.	R^2^	F
	C	1.590	0.185	8.580	0.000	0.122	5.655
*P_N_* (III)	*X*_14_	0.238	0.065	2.783	0.006 **		
	*X*_11_	0.225	0.036	2.641	0.009 **		
	*X*_10_	0.191	0.034	2.226	0.028 **		

Note: ** represents entering the model at a significance level of 5%.

## Data Availability

The datasets are available from the corresponding author upon reasonable request.
